# Illness perception of tuberculosis (TB) and health seeking practice among urban slum residents of Bangladesh: a qualitative study

**DOI:** 10.1186/1756-0500-7-572

**Published:** 2014-08-27

**Authors:** Kiran Bam, Lokesh Prasad Bhatt, Rajshree Thapa, Hussein Karimjee Dossajee, Mirak Raj Angdembe

**Affiliations:** James P Grant School of Public Health, BRAC University, Dhaka, Bangladesh; Institute of Medicine, Maharajgunj Medical Campus, Tribhuvan University, Kathmandu, Nepal

**Keywords:** Tuberculosis, Urban, Slum, Illness perception, Health seeking practice, Bangladesh

## Abstract

**Background:**

Combating tuberculosis (TB) in urban slums is more complex than in rural areas due to reasons such as over-crowding, unhygienic living conditions and poverty. This study aimed to assess illness perception of TB and identify barriers and facilitators for health seeking practice among the residents of Badda slum, Dhaka, Bangladesh.

**Methods:**

The Badda slum was purposively selected. Convenience sampling was carried out to select participants aged 18 years and above. Twenty two in-depth interviews, two key informants’ interviews and participatory rapid appraisal (PRA) were conducted. Data were analyzed manually by using defined a priori codes and color coding of the quotes in data matrix table.

**Results:**

TB was commonly recognized as *Jokkha* (pulmonary TB), *Sas rog* (disease associated to breathing) followed by TB. More females than males had knowledge about TB related illness. Very few perceived of being at risk of TB despite the high risk behavior and environment. Prime barriers for health seeking practice of TB were cost along with other barriers like prevailing stigma on TB, lack of information on service sites and unavailability of accompanying person. Training and orientation to community organizations and people, awareness on TB and free treatment through advertisements/media, community level diagnostic and home based care were identified as the facilitators for the health seeking practice of TB.

**Conclusions:**

Perceptions of TB and knowledge associated with the disease shape the health seeking practice, therefore promotion of media awareness campaign, targeting the people of urban slums for reducing misconceptions and promotion of home based service is needed to encourage health seeking practice in the future.

## Background

Bangladesh has the sixth highest rate of tuberculosis (TB) in the world with a prevalence of 411 cases per 100,000 population according to a report published by World Health Organization (WHO) in 2012 [1]; still the national case detection rate is 45% for all types of TB cases [2]. Dhaka, the capital city of Bangladesh is one of the fastest growing megacities in the world [3] with a population growth rate of 1.34% per annum [4]. High population density coupled with poor sanitation increase the spread of diseases including TB [5]. According to the case document published by Harvard business publishing on urban TB program in 2011 [6], combating TB in urban areas is more complex than in rural. Understandings on health and perceived severity of TB are vital factors for timely health care seeking and diagnosis [7]. Despite the availability of effective treatment and the healthcare providers giving their best efforts, most patients perceived that TB was incurable, and no TB patient could escape death. In some places TB was believed to be hereditary [8]. Majority of the people still rely on informal healthcare services and health care providers; and lack of knowledge on TB and traditional misbelieves were associated with delays in case detection [9]. The higher prevalence of TB among poor population indicates that the poor suffer more with delays in detection as well as treatment [10]. There is a paucity of published research on illness perception of TB and health seeking practices in urban slums of Bangladesh. This study aimed to assess the illness perception of TB and identify the facilitators and barriers of health seeking practice among the residents of Badda slum of Dhaka, Bangladesh.

## Methods

### Study area

Badda- a densely populated urban slum in Northern Dhaka, Bangladesh was chosen as the study area. Most of the inhabitants were migrants from different parts of Bangladesh.

### Inclusion and exclusion criteria

Male and female residents of Badda slum who were ≥18 years of age were included in the study. Those who were not able to provide consent and those who declined to answer the questions either due to their busy schedule or reluctance to participate were excluded. A total of twenty seven participants were approached for in-depth interview (IDI), out of which five (three females and two males) refused to participate in the study. Of those who declined to participate, two females were too shy to speak whereas the other three participants were busy with their own chores and chose not to participate.

### Study design and theoretical framework

**Figure 1:**
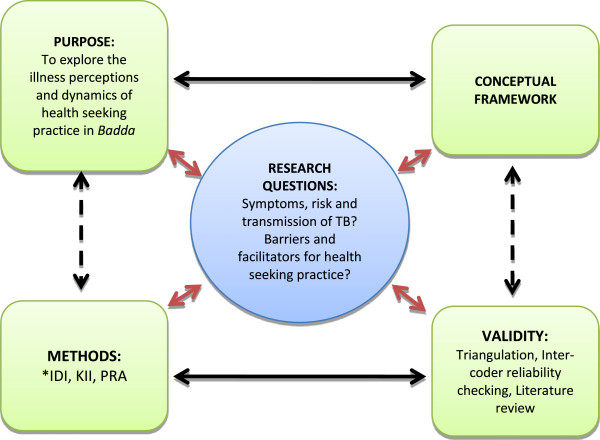
**Maxwell iterative model for illness perception of TB and health seeking practice.** *IDI- In-depth interview, KII- Key informant interview, PRA- Participatory rapid appraisal.

‘Realist theory’ was applied to clarify the research objectives and ground claims of knowledge, truth, progress and reality acquired through research [11]. Maxwell’s iterative model (Figure 1) was used to conceive the study where the research questions of the study correlate with the purpose, the conceptual framework and the validity of the study. The associated factors such as demographic variables, enabling factors and health service related factors were linked with illness perception, and health seeking practice.

### Data collection methods

Qualitative research methods are considered more suitable for exploring and defining the socio-cultural context of complex health behaviors [12].

- Twenty two IDI were carried out using semi structured interview guidelines. IDI were used in order to understand individual perception of TB and health seeking practice in detail. An equal number of male and female participants were selected in order to obtain a better understanding of the situation. The guideline was prepared and translated in to *Bangla* (local language) after discussion with the study team and implemented among participants by *Bangla* speakers. The questions inquired about the local terms related to TB, perceptions of the symptoms and transmission, perceived risks, health seeking practice including the barriers and facilitators for accessing TB treatment in the community.
- Participatory rapid appraisal (PRA) methods were used to identify the perception of illness among the individuals [13]. First, body mapping was carried out where body sample image of the front and back side was provided on a paper. Participants were then asked to mark or point on the image, the places where they perceived illness related to TB. In addition to that, participants were asked to list the barriers and facilitators for accessing TB treatment and rank them accordingly. Due to the difficulty in translating medical terminology into ‘*Bangla*’, this was deemed to be the best method of identifying regions of the body perceived to be affected by TB.
- Key informants’ interview (KII) was conducted with the service providers of urban TB center using interview guidelines. Questions related to illness perception, barriers and facilitators of health seeking practice were developed and asked in *Bangla* language. KII was used because it provided perceptions of the health care providers and enabled comparison with data obtained from the community through IDI and PRA.

The semi-structured interview guidelines consisted of open-ended questions to let participants describe their personal opinions, perceptions, and experiences. The individual interview was audio recorded with the participants’ consent. All interviews were conducted in a room that facilitated private discussion. Participants’ observations were noted at the same time. The interviews lasted between 5-6 minutes in case of IDI and 35-40 minutes in case of PRA exercise. KII and IDI were stopped as soon as saturation of information was reached.

### Sampling

We selected Badda slum purposively due to ease of access during the ongoing political instability in Dhaka. Convenience sampling was carried out for selection of participants because of difficulty in obtaining appointments in advance. Participants were selected in such a way so as to ensure equal number of males and females and the recruitment continued until data saturation was met and no further new information was obtained. We conducted twenty two IDI with eleven males and eleven females, two KII and one PRA in coordination with the health professional from BRAC (an NGO) urban TB center of Badda. Details of the participants’ characteristics are shown in Table 1.

**Table 1:** **Participants’ socio-demographic characteristics**

Socio demographic characteristics	Number (%)
**Gender**	
Male	11 (50.0)
Female	11 (50.0)
**Age** ^**a**^	
≤35	15 (68.2)
>35	7 (31.8)
**Occupational status**	
Restaurant worker	1 (4.5)
Driver	4 (18.2)
Garment worker	4 (18.2)
Housewife	11 (50.0)
Shopkeeper	1 (4.5)
Ticket seller	1 (4.5)
**Educational status**	
Literate	19 (86.4)
Illiterate	3 (13.6)

Age, occupational status and educational status were not grouped by gender.

^**a**^For breakdown of age median values were used.

### Data analysis

Each interview was audio-recorded with the participant’s permission and field notes were taken for all the interviews. Nine inductive a priori codes were identified and defined. Transcription was carried by *Bangla* speakers, inter and intra coder reliability check was calculated at >90%. Manual color coding and sub-coding of each interview in MS Word 2007 was done. Similarly, common and similar issues were identified and analyzed following qualitative data analysis process [14]. Data triangulation was done by comparing findings from IDI and PRA.

### Ethical issues

Approval for the study was obtained from the ethical review board at James P Grant School of Public Health, BRAC University, Dhaka, Bangladesh, and BRAC urban TB center, Badda. Participants provided verbal informed consent before the beginning of interviews and before audio recording. Confidentiality was maintained and each participant was duly informed about research objectives, potential risks, and benefits prior to the interview. Participants agreed to the use of data, provided that anonymity was maintained. Pseudonyms were created where verbatim quotations were used.

## Results and discussion

The mean age of participants was 33 years (age range: 20–65 years). The socio-demographic characteristics of participants are shown in Table 1.

### Local terms for TB

During PRA discussion, most of the participants perceived TB as a serious disease and cited different local names for TB. The majority of the participants mentioned *Jokkha* (pulmonary TB), *Sas rog* (disease related to breathing) followed by ‘TB’ as the commonly used names in the community. However, some of the participants also considered it as cancer.

*“We call it cancer [chronic disease] or ‘Jokkha’, we learnt this from our parents. Most of us use ‘Jokkha’ in our society, some people call it ‘sas rog’ as well”*. (IDI2, LN 34)

*“TB is the disease where there is cough with blood. We used to call it “Jokkha”, now it’s called TB”.* (IDI6, LN 58-59)

### Illness perception of TB

Perception of the symptoms, risks and transmission of TB were assessed during this study through body mapping. Still, there were some misconceptions related to TB like the belief that TB is characterized by appearance of blood in urine and TB and cancer are synonymous. In terms of symptom perception, most of the participants recognized cough, appearance of cough with blood, weight loss, and weakness as TB symptoms.

*“I suffered from fever and weight loss for a long time. I was a little chubby and healthy, but now I am shrunken and I don’t feel easy to work”.* (IDI4 LN 68-69)

Majority of those who sought health care reported cough as a chief health complain. This finding is similar to a study carried out in China [15]. However, the participants were unaware that it might be due to TB.

*“Most of the people come here with symptoms of cough. For those having cough, we ask them for how long they have been coughing ?, most of them say one week to two weeks with chest pain, fever, and loss of appetite”.* (KII, LN 2-4).

More females than males mentioned the correct symptoms of TB, especially older females. These findings are contrary to that of a study in Vietnam where more males perceived the symptoms than females [16]. Females were more likely to seek health care services than males as males are more likely to overlook these symptoms as being negligible [17].

Very few IDI participants perceived themselves at risk of getting TB, despite their risky lifestyle, and environment. The low risk perception among the respondents can ultimately lead to delay in health care seeking [18].

*“No, we don’t have any chances of getting TB, we are living in a healthy place, we eat healthy and our daily practices are also healthy.”* (IDI6, LN 82)

*“In the area where I reside, the risk of getting TB is low; nobody is infected with TB as far I know”.* (IDI 7, LN 51-52)

When we asked *what would you do if you think you have TB?* Most of the participants were aware on the availability of TB treatment. It was found that TB treatment has been readily available in recent days. Previously, TB was a scary disease but with the availability of health personnel in the community, people are aware that it can be cured and hence they are fear less.

*“The environment is now better in the community; good doctors and treatment is available; now a days we can get diagnosis and treatment facilities as early as possible…we are happy enough.”* (IDI 18, LN 49-51)

### Health seeking practice and associated factors

When ill, most of the participants seek modern medical care such as allopathic pharmacies, diagnostic centers or hospitals. Others also reported to seek traditional care and homeopathic care. Pluralistic health seeking practice as described here has also been documented elsewhere [18].

The major reported barriers to health seeking were lack of money (cost), fear from treatment procedure; fear of stigma, unavailability of accompanying person, time constraints, and lack of information about treatment facilities. Financial problem was listed as the top most factor hindering health care seeking practice during free list ranking. This is similar to the findings by Xu et al. (2004) in China [19] which found financial difficulties to be the major factor for delay in health care seeking.

*“Some may not go due to his/her financial problems. This is the number one cause. Otherwise everybody would seek treatment after knowing about the disease. The only reason why a person denies medical care is because of financial constraint”.* (IDI 20, LN 86-87)

Stigma related to TB still exists in the urban slums which have led to delays in health care seeking. Similar reasons were found in a study carried out in North Ethiopia where stigma had influenced low case detection and delays in health care seeking [20]. A study in India in 2009 showed the persistence of immense stigma related to TB observed at society level with 60% of the patients hiding their disease [21]. Similarly, stigma related to TB has been evidenced to be significant for delay in TB treatment [22].

*“Whenever anyone gets TB, other people keep distance from them. No one sits beside them. They keep distance. There was one among us but it was only after a while that we knew that he was suffering from TB. We found this out after observing the behavior of his relatives and family members towards him”.* (IDI 16, LN 96-97)

“*In early days our grandparents told us that most of the people suffer from unknown disease [undiagnosed] and there was this conception that man who gets TB [diagnosed] has to die”.* (IDI12, LN 61-62)

Comparatively, health seeking practices have improved over the years which may be accounted to regular awareness programs among community members and improved community level diagnostics and home based care. Group ranking exercise identified advertisements/media as the most important facilitator for seeking health care which is similar to the finding from India [23].

The key informants identified community level care as an integral part of TB diagnosis. The services were made accessible through the smear centers at slums however there are still information gaps regarding the sputum collection at the community level.

“*Service should be as close to the people as possible and with this aim out- smear center has been established which disseminates information regarding sputum collection usually a day prior to sputum collection. On the very day sputum collection box is distributed and the sputum is collected the other day. Sputum collection is carried out twice a week, usually on Sundays and Tuesdays”.* (KII2, LN 92-97)

*“….no any idea about the facilities around or the treatment available, we don’t know what actually we have to do”.* (IDI9, LN 84-86)

According to IDI participants there is home based, directly observed treatment short course (DOTS) facility in the community which helps them to access services easily.

“*In my village, there are shebikas (female volunteer care takers). They bring medicines from BRAC, and make people take it every morning”.* (IDI3, LN 145-46)

This study had several limitations. Since the participants had to remember past events, there was a possibility of recall bias. This was minimized by asking questions to the participants more than once and reconfirming every response. Interviews were conducted in ‘*Bangla*’ and translated in to English. In this process the verbatim meaning of answers may have been lost. Majority of the participants were literate (86%) and 35 years of age or younger (68%), thus the results may be underrepresented for those who are illiterate and older than 35 years. Despite Badda slum representing a population that is poor and overcrowded, its residents are highly migratory and may not be representative of urban poor populations within Dhaka city.

## Conclusions

Every participant had some knowledge about TB but it was not comprehensive. Majority of the participants perceived themselves to be at medium or low risk of TB. Perceptions of TB and knowledge associated with the disease shape the health seeking practice. Major barriers to care-seeking from health facilities includes: lack of money for transport and treatment. In case of treatment, although DOTS is free of cost, expenses such as cost paid to local pharmacist for drugs other than DOTS as well as the cost of diagnostic tests add to the economic burdens of ultra-poor slum dwellers. These barriers can be reduced by bringing the health services closer to the community, carrying out community sensitization programs about TB including adult literacy programs. Advertisements/media was the most important facilitator for health seeking practice. Thus promotion of information, education and communication (IEC) materials which are culturally sensitive may lead to behavior change among those who are at constant risk of TB. Further research on illness perception of TB and health seeking practice in urban slums is required to increase understanding of this public health problem.

### RATS guidelines

This study follows the RATS guidelines on qualitative research.
